# Understanding the support experiences of families of children with autism and sensory processing difficulties: A qualitative study

**DOI:** 10.1111/hex.13465

**Published:** 2022-03-18

**Authors:** Sarah Milosevic, Lucy Brookes‐Howell, Elizabeth Randell, Rhys Williams‐Thomas, Sue Delport, Monica Busse, David Gillespie, Alka S. Ahuja, Anne Marie McKigney, Eleni Glarou, Rachel McNamara

**Affiliations:** ^1^ Centre for Trials Research Cardiff University Cardiff UK; ^2^ School of Healthcare Sciences Cardiff University Cardiff UK; ^3^ Aneurin Bevan University Health Board Newport UK; ^4^ School of Medicine Cardiff University Cardiff UK

**Keywords:** autism, children, parents, qualitative research, sensory processing, support

## Abstract

**Background:**

Support, such as information, advice and therapies, can play a vital role in the lives of families of autistic children. However, little is known about the support experiences of UK parents and carers.

**Aim:**

To explore experiences of and access to support for families of children with autism and sensory processing difficulties, from the perspective of parents and carers.

**Methods:**

Semi‐structured, timeline‐assisted interviews were conducted with parents/carers of 30 children aged 5–11, exploring experiences of support. Framework analysis was used to identify themes in the interview data.

**Results:**

Support varied widely and was not accessed equitably. Specialist autism support, together with support from other parents and voluntary organizations, was perceived as more useful than statutory and nonspecialist provision. Unmet support needs included an ongoing point of contact for information and advice for parents, and access to direct therapy and specialist mental health provision for children.

**Conclusions:**

Findings emphasize the need for a clear pathway of support following autism diagnosis, autism‐specific training for professional service providers and specialist provision tailored to the needs of autistic children.

**Patient or Public Contribution:**

An advisory group of four parents of children with autism provided feedback on study procedures and materials, including participant information sheets and timeline completion instructions.

## 1 BACKGROUND

It is estimated that 1%–2% of primary school children in the United Kingdom have autism.^1^ Autism can have a substantial impact on school and family life, and the estimated annual cost of formal and informal support is £3.1 billion.^2^ Therefore, it is vital that support is effective and delivers value for money. For the purpose of this study, ‘support’ is defined as encompassing information, advice, support groups and therapies for children with autism and their parents, provided via statutory services, charities or informal networks. A recent report highlights significant gaps in the evidence base for autism support.^3^ Services across the United Kingdom are variable,^3^ and research suggest there is no defined pathway for support of children and parents following autism diagnosis.^4^ There is a clear need to further explore support experiences amongst this group, focusing on both access to support and the usefulness of provision.

Children and young people with autism are more likely than the general population to experience anxiety,^5^ bullying^6^ and a lack of friendships,^7^ and are at greater risk of self‐harm behaviours and suicide.^8^, ^9^ There are also effects on the wider family, with parents of children with autism experiencing on average higher levels of stress and depression,^10^, ^11^ feelings of stigma^12^ and loss of earnings.^2^ Over 90% of children with autism also experience some sensory processing difficulties,^13^ which have been found to limit participation in daily life and leisure activities.^14^, ^15^ Hyper‐ and hypo‐responsiveness to sensory stimuli amongst children with autism are also associated with greater caregiver strain;^16^ therefore, parents of children with autism and sensory processing difficulties may require additional support.

Effective provision of support for families can mediate the effects of some of the aforementioned challenges. For example, professional support has been shown to reduce parental stress,^17^ improve parental competence^18^ and increase children's participation in daily life.^19^ Conversely, inadequate support can have negative consequences, such as feelings of isolation and alienation,^20^ lower reported psychological wellbeing^21^ and reduced parental participation in employment and leisure.^22^

Previous research has shown considerable variation in access to support (e.g., from psychologists, psychiatrists and speech and language therapists) for children with autism and their families.^23^ Provision in the United Kingdom varies across the devolved nations and by locality, due to differences in service availability, eligibility criteria, waiting times and local authority spending.^24^ A study of parents in London and the East of England found that while some felt well supported by a range of external organizations, others did not feel they had adequate support or information.^25^ Although little UK research has examined differences in provision between rural and urban areas, studies in the United States and Canada report that families living rurally experience considerably greater difficulty accessing specialized services.^26^, ^27^

Despite the wide range of autism support services available throughout the United Kingdom, little contemporary research has explored the relative perceived usefulness of different forms and sources of support. Parents surveyed in one county in England quantitatively rated support received from early years courses and their child's school as most useful, together with information from family workers, local support groups and workshops.^28^ An interview study of 20 parents in London and the East of England found support from other parents of children with autism was highly valued, enabling the sharing of ideas; charities were also cited as providing helpful information.^25^ However, this study was conducted over a decade ago in a discrete geographical area, and it is proposed that further qualitative research is needed to add validity to the evidence base.^3^

Given the challenges faced by families of children with autism—and the additional support needs where children also have sensory processing difficulties—effective provision for this group is of vital importance. There is currently limited knowledge of the support experiences of UK families, and little research to date has explored the utility of existing interventions. This study aimed to qualitatively explore access to and perceived usefulness of support for families of children with autism and sensory processing difficulties, from the perspective of parents and carers.

## 2 METHODS

### 2.1 Study design

This qualitative study was conducted as part of a randomized controlled trial of sensory integration therapy plus usual care versus usual care alone for children with autism and sensory processing difficulties (the SenITA study).^29^ Children in the sensory integration therapy group received 24 face‐to‐face sessions with occupational therapists, participating in play‐based sensorimotor activities. The study was approved by Wales Research Ethics Committee 3 (reference 17/WA/0031). Informed consent was obtained from all participants.

Semi‐structured interviews were conducted with parents/carers of children enroled in the SenITA study. To enhance the richness of interview data, interviewees were asked before the interview to create a timeline of key events in their child's life, which they used as a tool to share their experiences with the researcher. Chronological visual methods such as timelines can help interviewees to structure a narrative account that reflects the breadth and depth of their experiences, which may not be fully elicited via traditional interview methods.^30^

### 2.2 Participants

Interview participants were parents or carers of 30 children with autism and sensory processing difficulties enroled in the SenITA study (see Table 1), recruited from three areas of England and Wales: South/West Wales, Cornwall and North Buckinghamshire. Inclusion criteria for the SenITA study were that children had to: (1) have a diagnosis of autism documented in their medical/educational records, or be undergoing assessment within the local autism diagnostic pathway; (2) be aged 4–11 years; (3) be in mainstream education and (4) have sensory processing difficulties (definite dysfunction on one or more sensory dimensions and the total score of the Sensory Processing Measure,^31^ or probable dysfunction on more than one sensory dimension and the total score of the Sensory Processing Measure). Those receiving or who had previously received sensory integration therapy, or who were receiving an intensive applied behaviour analysis‐based intervention were excluded.^29^

**Table 1:** Participant characteristics

Characteristic	No.
Gender of child	
Male	23
Female	7
Age group of child	
5–6 years	6
7–8 years	9
9–10 years	8
11 years	7
Ethnicity of child^a^	
White British	23
Mixed ethnic background	2
White other	2
Study region	
Wales	20
Cornwall	6
North Buckinghamshire	4
Rurality^b^	
Urban	23
Rural	7
Relative deprivation^c^	
50% Most deprived	18
50% Least deprived	12

^a^ Ethnicity was not disclosed for three children.

^b^ As assessed by the Rural–Urban Classification 2011.^32^

^c^ Relative deprivation is calculated separately in England and Wales,^33^, ^34^ so is not directly comparable.

Most interviews (26) were conducted with children's mothers, two were conducted with mother–father pairs, one with the child's father and one with a grandparent. At the time of interview, children were aged between 5 and 11 years (mean: 8, SD: 2.0).

### 2.3 Materials and procedure

Interview participants were recruited purposively from the SenITA study database to ensure variation as far as possible in terms of child gender, age and geographical location. Recruitment ceased when the depth and breadth of data collected were considered sufficient to capture the range and richness of participants' experiences. Potential participants were contacted by the qualitative researcher via telephone or email and asked whether they would be interested in taking part in an interview. If they agreed, they were posted an information pack containing a participant information sheet, two copies of a template timeline, timeline completion instructions with an example completed timeline and some coloured pens. Around 1 week after posting the information pack, the qualitative researcher recontacted parents/carers to ask whether they were willing to take part. If they agreed, a mutually convenient time for the interview was arranged. Participants were given the option of taking part in an interview face‐to‐face at their home or workplace, or over the telephone. They were offered a £10 shopping voucher as a thank you for their time.

Interview questions (see Table 2) were developed by qualitative researchers S. M. and L. B. H. in collaboration with the SenITA study management group and parent advisory group. There were eight main questions designed to explore participants' support experiences, each with several follow‐up questions to be used as prompts where more in‐depth information was needed. Interview participants were asked to complete the template timeline before the interview; those participating in telephone interviews were asked to send a copy of the timeline to the researcher via email. The timeline instructions requested parents/carers to note down the different types of therapies, interventions and strategies they had experienced with their child, from the time they first saw signs that their child might be autistic until the present day.

**Table 2:** Interview questions

Question number	Question
1	Could you talk me through the timeline? (If completed)
2	Could you tell me a bit about your child's sensory problems?
3	Have there been any key events or significant people involved in the support you and your child have had for their autism?
4	Could you tell me about the therapies or interventions your child has had for his/her autism? Have any of these been particularly useful/not useful?
5	Could you tell me about any strategies, ideas or advice you have been given in relation to your child's autism? Have any been particularly useful/not useful?
6	Are there any therapies, interventions or strategies that you tried for a very short time and then stopped? What were the reasons?
7	Are there any particular therapies, interventions or strategies that you have tried that you would recommend to someone else?
8	Have you encouraged your child to participate in any activities or groups to help them?

Interviews were conducted between February 2019 and March 2020 by S. M., an experienced qualitative health researcher not involved in the provision of autism services. Most interviews (19) were carried out face‐to‐face, with 11 conducted over the telephone. For face‐to‐face interviews, participants completed a written consent form before the commencement of the interview. Consent for telephone interviews was obtained verbally and audio‐recorded. Interviews were between 12 and 86 min in duration (mean: 38.0, SD: 17.6). All interviews were audio‐recorded using a digital voice recorder, with the permission of the participant. Just under half (13) of participants completed the timeline of key events in their child's life; the remainder reported they had forgotten or not had time to complete this.

### 2.4 Patient or public contribution

Parents of children with autism were involved in the design of the study to inform the development of study recruitment and data collection procedures, helping to ensure suitability for the participant group. An advisory group of four parents was formed, with members providing feedback via face‐to‐face meetings and email. Contributions to study design included suggestions of groups and charities that could be targeted to advertise the study to parents and health professionals, and feedback on qualitative data collection materials. For example, it was suggested that timeline instructions should include specific questions to help parents recall more detailed information about their support experiences. A list of prompt questions was subsequently devised and used successfully. Due to time constraints, the advisory group was not involved in the analysis of qualitative study data, which could have provided additional insight into study findings and would be useful to incorporate in future studies.

### 2.5 Analysis

All interviews were professionally transcribed verbatim and anonymized. Two qualitative researchers (S. M. and L. B. H.) analysed the transcripts using a framework approach.^32^ Framework analysis has five key stages: (1) familiarization with the data, (2) identifying a thematic framework, (3) indexing (coding) the data, (4) charting by topic or theme and (5) mapping and interpreting the data set. In accordance with the research aims, and after a preliminary review of interview transcripts, three key themes were identified for further exploration: support provision, usefulness of support and gaps in support. S. M. then read through interview transcripts and compiled a list of subthemes emerging from the data. Following discussion with L. B. H., S. M. developed an analytic framework and used this to code interview data in NVivo version 12 (QSR International). To enhance the trustworthiness of the analysis, L. B. H. independently coded 10% of interview transcripts, with any disagreements in coding resolved through discussion. S. M. compiled tables to summarize each participant's experiences in relation to the themes and subthemes. Illustrative quotes from the coded transcripts were included so that the validity of themes could be reviewed by both qualitative researchers to ensure they accurately reflected the experiences of participants. The themes were discussed until consensus was reached.

## 3 RESULTS

**Figure 1:**
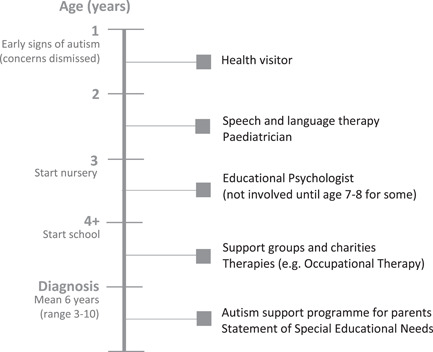
Typical timeline of support accessed by families

Completed timelines enabled examination of the support families typically accessed at each stage of their child's journey (see Figure 1). Although experiences varied widely, the involvement of key professionals in the early stages tended to follow a similar pattern. Parents described noticing the first signs of autism when their child was aged 1–2 years. At that stage, the families' key contact was the health visitor, with whom parents raised initial concerns (which were often dismissed). Difficulties with speech tended to be noticed by professionals early on, leading to the involvement of speech and language therapists and paediatricians. Starting nursery and then school appeared to be a causal factor in support provision increasing, including the involvement of charities and professionals such as occupational therapists and psychologists. However, most children did not receive any specialist autism support until later in their primary school life. Some forms of support, such as the National Autistic Society EarlyBird Programme (a specialist autism support programme for parents), were not accessed until after children had received a diagnosis. Following diagnosis, service provision was much more varied, and other than the EarlyBird Programme, there was no obvious pathway to support.

**Figure 2:**
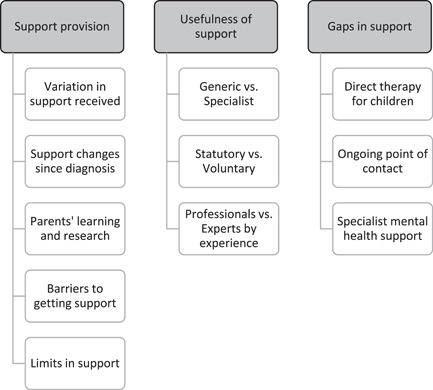
Themes and subthemes

Following analysis of timelines and interview transcripts, three main themes were identified as representing the key perspectives provided by the data. Within each main theme, there were several emergent subthemes (see Figure 2). These are discussed in turn below, illustrated by quotes from participants. All participant quotes are labelled with a participant ID number to preserve anonymity.

### 3.1 Support provision

#### 3.1.1 Variation in support received

There was wide variation in the support received by families, with some accessing between one and three sources of support, while others were supported by 10 or more organizations or professionals. There did not appear to be any differences in the amount of support accessed in relation to child age, gender or rurality, although some parents reported limited local service provision. There was no clear pathway to support, which was accessed in multiple ways, including via school, parents' own research and networks, medical professionals and charities. Often there was a domino effect, whereby families were referred into one service, which referred them to another, and so on.

So the health visitors suggested Camau Bach [an early intervention disability support service] and then SNAP Cymru was [suggested by] Camau Bach and then the National Autistic Society was one that I found myself on Facebook.

(P2)

While one parent reported that services seemed to be provided automatically following their child's diagnosis, others found they had to be proactive in seeking support.

I went off and did ASD parenting courses, as you do. And probably went into doing quite a lot of self‐led learning and finding support groups for me and for the children… Everything we've done, we've done ourselves.

(P23)

#### 3.1.2 Support changes since diagnosis

Receiving an autism diagnosis enabled some children to access additional support, most notably in school; for example, by receiving a statement of special educational needs (a legal document detailing the educational needs of an individual child and how these will be met) or gaining a place at a specialist school. However, the effect of diagnosis varied, with some families continuing to receive the same level of support as they had before diagnosis and others accessing no support.

She had an official diagnosis of being autistic, and she was referred to the Autism Outreach Service… we attended the EarlyBird course… she received her statement of special educational needs and went full time to… a specialist school.

(P6)

They just give you a book… there's nothing, there's no therapy available or anything… I thought they'd be like, he's diagnosed, here are some therapies you can access… The only thing the assessment changed is my peace of mind that it's not me, it's not my parenting… but that was the only [benefit] because we haven't got access to anything extra really… it's pointless, well it's hard.

(P13)

#### 3.1.3 Parents' learning and research

Although diagnosis did not always result in increased support provision, parents reported that it enabled them to seek resources for themselves. This resulted in enhanced understanding of their child's behaviour and sensory processing needs, and the ability to support them appropriately.

When we started to go down the ASD route, I threw myself at every available bit of training… and that really helped… The biggest thing it gave me was just to drop all the ‘shoulds’. You know, I thought as a ten, eleven‐year‐old boy you should be able to wash your hands. You should be able to brush your teeth… It completely allowed all those ‘shoulds’ to dissolve, and what was left was just enquiry really. Okay, why is this hard, what can we do to support you?… It revolutionised our family life to be honest. From nagging and moaning and just frustration, to oh okay, you know, your brain works a bit differently, so we need to do different stuff.

(P3)

Some people say why would you want to put a label on it, it's not about putting a label on her, but we didn't know if we were doing things correctly… because obviously looking back now, some of the things that we were doing wasn't helping, it was making things worse.

(P16)

Following diagnosis, many parents had joined support groups and embarked on self‐led learning. However, this was dependent on their personal circumstances, networks and finances. For example, some had paid for support, others had accessed courses via their own employment or voluntary roles, and some had gained information or advice from friends who were professionals in the field.

#### 3.1.4 Barriers to getting support

A key barrier to accessing support was the dismissal of children's difficulties by professionals, resulting in delays in diagnosis, rejection of referrals for additional support, and needs not being provided for in school. For example, several parents described noticing signs of autism when their child was a toddler, but waiting years to receive a diagnosis. This was sometimes due to children masking outside the home, meaning behaviours indicative of autism were missed.

Throughout [her] schooling they've never had any concerns… [never] any suspicions of ASD, but she's an incredibly able masker.

(P28)

Although schools were an important source of support for some families, in several cases there was a lack of understanding of children's sensory needs, which were not recognized or addressed consistently.

At school he has a very strict rigid sensory timetable… six sensory breaks throughout the day… [but] when… he's good for a couple of weeks… they slack on [it]… and then he'll start to kick off. I'm like, ‘Well, are you doing all the sensory stuff?’ ‘Oh no, because he's been really good’.

(P25)

[The occupational therapist] tried her with [a] wobble cushion… [which] she benefitted from. Bought a wobble cushion, took it to school, ‘What the hell is that?’ They weren't very up for using it… she just got told you're a big girl, you can sit still. And so I… had the argument of, but if she's concentrating so much on sitting still then she's not actually receptive to what you're teaching her.

(P28)

#### 3.1.5 Limits of support

In addition to gaps in support provision, the support families did receive seemed to be time‐limited or ended following diagnosis. Where support had been helpful, this was confusing for children and frustrating for parents.

I find the support have stopped… [the] Occupational Therapist has gone, it's like everyone's gone now. So we've got no support… [and] he's still struggling every day.

(P14)

He's had so many like different departments involved in his care… Now… it's like right… we've helped you… we can't do no more… so basically everybody's signed him off. He's confused because he now thinks that everyone's just dropped him and doesn't care.

(P17)

Resource limitations (such as helpful sources of support being under‐funded or over‐subscribed) meant some families faced long waiting lists for services or could not afford the support their child needed.

I've looked into private SI [sensory integration therapy], but… it's stupidly expensive… it's like £50 odd a session. I mean, I can't afford that.

(P13)

There's an EarlyBird programme for parents of children with autism, but it's like a year's waiting list. And then I was offered one and I was either away or I couldn't get out of work, and I said is there another date at all, I really can't go to this. They went no, go away, another year or something.

(P18)

Some parents mentioned a lack of services locally, meaning families had no access to certain forms of support, or had to travel to receive it.

We haven't had a huge amount of involvement because there isn't a huge amount of resources in… Cornwall.

(P25)

It's very hard in Cornwall to get onto any parenting course that's in the evening, and [the courses are far away, so] I have to either catch the ferry… or drive the hour round trip, all around the coast… It's not practical and it, it puts you off, you think… what's the point, and… try and battle on.

(P24)

### 3.2 Usefulness of support

#### 3.2.1 Generic versus specialist

Parents viewed specialist support as being most useful, while more generic support often did not meet the needs of the child or family. Health visitors tended to be parents' first source of help and advice (and were gatekeepers to specialist services) but were mostly described as being dismissive of the early signs of autism.

I raised issues about speech quite early on. And [the health visitor] said oh he'll get it, he'll get it. They just constantly kind of brushed it aside.

(P25)

He passed these very basic tests and [the health visitor] was like there's nothing to worry about, there's nothing to worry about, he's a bright boy… [It] just seemed like no‐one was listening.

(P18)

Similarly, parents reported that Speech and Language Therapy (SALT) and Child and Adolescent Mental Health Services (CAMHS) did not appear able to provide appropriate support to their child.

We've seen Speech and Language, because he stammers, and he seems to have selective mutism… but they just let us go because he wouldn't speak in… the appointment and his understanding was fine.

(P13)

[CAMHS are] just… not equipped to deal with autism… she's got severe, severe anxiety… but she's not being treated for it because they don't know how… We did a bit of work with one lady, after four weeks, she said ‘Oh I'm unable to help her… she just doesn't understand it’… Meanwhile… it's getting a whole lot worse and having a massive impact on her life.

(P8)

In contrast, specialist support was viewed as very helpful, particularly autism‐focused parenting courses and advice from specialist occupational therapists, for example, in relation to sensory processing difficulties that affected children's daily routine.

I've been on loads and loads of [parenting] training… the only course I can honestly say that I did enjoy was the EarlyBird course. Now… that was a great help… more realistic… I really got on with the parents there, they were in the same situation as me and that's what I wanted… it opened my eyes, I understand, understood a lot more.

(P16)

#### 3.2.2 Statutory versus voluntary

Unlike statutory services (such as health visiting and SALT), charities and local disability groups were seen by parents as being the most helpful forms of support, even where these were not specific to autism or sensory processing difficulties. They appeared to fill some of the gaps in statutory provision and provided much‐needed help and advice to families.

[A local] charity helping disabled children [learn] through play… has been a lifeline… [The psychologist] is able to talk to him with the action figures, or with the Lego… they can talk about feelings through that, which is a great asset because… empathy was really hard for him. He really struggled.

(P9)

Disability groups also enabled children to participate in extracurricular activities (such as swimming, soft play and cinema), which they typically found difficult to access. Parents described these as providing a less pressured environment than mainstream activity groups.

Once a month they do… soft play, trampolining and things like that, and that was good because [she] was a different child in that. So I think perhaps she sensed… that everybody was the same… And it was nice because I didn't have to think, oh god, is she going to kick off… because I do get anxious when we're out.

(P16)

#### 3.2.3 Professionals versus experts by experience

As previously stated, parents reported that professionals often lacked expertize in autism, and were unable to provide support tailored to the child's needs. They were described in some cases as being dismissive or judgemental.

You see it time and time again, it's schools being unsupportive. Professionals saying, ‘There's absolutely nothing wrong’, even though it's staring them in the face. It's the parent, you know, the parental blame… your parenting skills.

(P29)

She was very negative… towards [our] parenting… And I actually spoke to CAMHS after all this and said, ‘I don't want her coming back’, because… she made me, well she made us both feel like [we] haven't done enough really.

(P24)

In contrast, online and face‐to‐face contact with other parents of children with autism was valuable in enabling the sharing of strategies and ideas and providing nonjudgemental support and understanding.

I am with a lot of forums… I can ask them anything… Talking to other parents is brilliant… they're probably the best [source of] advice.

(P11)

You know that somewhere [on the Facebook group] there'll be… someone saying, yeah I hear what you're saying, I've been there, I get it, you're not the only one. And that can be a massive thing, because obviously being a parent of a child who's got extra needs, it can feel quite lonely… and it's nice to know that someone actually understands.

(P29)

### 3.3 Gaps in support

#### 3.3.1 Direct therapy for children

An important gap in provision related to direct therapeutic support for children, particularly occupational therapy for sensory issues. Some parents identified that most services their family had accessed were focused on the parents rather than the child, or on diagnosis rather than support.

The integrated autism service… have an OT [Occupational Therapist] and all sorts but they don't actually see the child, they just speak to the parents… People give me advice but it's easy for them to say do x, y and z… we need like hands on [support].

(P13)

[Services have been] mostly about diagnosing. As opposed to helping.

(P21)

#### 3.3.2 Ongoing point of contact

Parents highlighted a need for a key ongoing point of contact, who could signpost to services or help them deal with new issues arising. For a number of families, school was their main or only continuing source of support.

What I find now… if there's an issue that crops up, because [three sources of support] have all finished, [I've] only really got the school to deal with now, so if I have an issue, I have got I suppose the Autistic Society… but I find I haven't got anyone… like a professional I can actually ask… you know, what could I do to help this sort of situation?

(P2)

#### 3.3.3 Specialist mental health support

Specifically, there was a vital unmet need for appropriate mental health support for children. In some cases, this was because families experienced difficulties accessing services, as there appeared to be a high threshold for referral. For those who did gain access, support was very limited or unhelpful.

There's no help… for these children at all… I think it took six referrals to CAMHS… she grabbed a knife, tried to chop her own head off… they weren't really worried about it… how bad do things have to get before an intervention comes?

(P8)

CAMHS sort of fell off the edge of the earth… ‘Well there's nothing more we can do’.

(P24)

## 4 DISCUSSION

This study qualitatively explored experiences of support for families of children with autism and sensory processing difficulties, from the perspective of parents and carers. We found wide variation in support provision, with no clear pathway to support following diagnosis, and access depending to some extent on parents' own learning and research. Unlike specialist autism provision (which children typically accessed relatively late), generic support tended not to meet the needs of families. The dismissal of children's needs by professionals was a key barrier to families accessing support, along with resource limitations, such as long waiting lists or a lack of local services. Parents identified unmet support needs, including the need for an ongoing point of contact for information and advice, and direct therapy and specialist mental health support for their child. The need for health professionals to provide nonjudgemental support and understanding was highlighted; peer support was described as particularly useful in filling this gap. Findings build on limited knowledge of the support experiences of families of children with autism and provide new evidence on the perceived usefulness of varied forms of support.

Findings align with data showing that service provision for children with autism is variable across the United Kingdom.^24^ Access to existing provision has previously been found to depend on socioeconomic and family circumstances.^23^, ^25^ For example, families of children with Down syndrome—who tend to report a lower incidence of poverty and higher levels of parental education compared to families of children with other neurodevelopmental disabilities^36^, ^37^ are more likely to report having their support needs met.^38^, ^39^ We found that in some cases, autism support was accessed by families as a result of parents' professional or social networks. Some had the ability to proactively seek out sources of support or information, or to pay to access services privately, while this was not an option for others. The lack of a defined pathway of support for families of children with autism (as found in this study and previously)^4^ is likely to compound this disparity.

Specialist rather than generic support was described as most helpful to families in this study, consistent with previous survey findings that parents rated information provided by an autism family services worker as more useful than information from professionals not specialized in autism.^28^ Parents in the present study explained that services such as SALT and CAMHS did not seem equipped to support children with autism, discharging them due to assessment difficulties or an inability to address their specific needs. In support of this, an international systematic review found that limited knowledge of autism amongst therapists was a key barrier to mental health support for children and adults with autism.^40^

Support from charities filled an important gap in provision and tended to be seen by parents as more useful than statutory services. Consistent with previous research,^14^ parents reported that accessing leisure activities was challenging due to their child's sensory difficulties. Therefore, local disability events were particularly valued in enabling families to be part of a social group. For parents themselves, being able to socialize face‐to‐face or online with others in a similar situation was an important source of support and advice. As found previously,^28^ parents appeared to find support groups more useful than advice from professionals, who often lacked autism expertize or understanding of their situation. Furthermore, contrary to the suggestion that parents of children with autism no longer experience blame from professionals,^41^ parents in this study described feeling judged as their child's behaviour was blamed on their parenting skills, indicating a learning need for professionals. In contrast, parent support groups provided a nonjudgemental atmosphere where parents reported receiving support and understanding. As parents of children with autism report experiencing higher levels of stigma than those caring for children with other (neurodevelopmental or physical) disabilities,^42^ this nonjudgemental support is likely to be of particular importance.

The main barrier to families accessing support was the dismissal of their child's difficulties by a range of professionals. Given that all children were in mainstream education at the start of the SenITA study, they may have experienced less severe difficulties than those educated in specialist settings, making it more likely that subtle signs of autism could be missed. As commonly experienced in the United Kingdom,^43^ some parents reported having to wait several years between first expressing their concerns to a professional and receiving a diagnosis for their child. Some children masked their symptoms outside the home, meaning that professionals did not always see or recognize behaviours indicative of autism. As found previously,^44^ this led to delays in diagnosis and support provision. Diagnostic delays may partially explain the disparity in support received by families of children with autism and those of children with Down syndrome for example, who typically report fewer barriers to accessing services and greater social support.^38^, ^39^

Surveys of parents of children with autism have previously identified broad unmet needs, relating to respite care, information about services and advice.^23^, ^45^ Interestingly, although parents in this study were not directly asked about unmet needs, these were raised by most interviewees, and findings highlight three key gaps in support. In contrast to the results of previous surveys, these are relatively specific: direct therapy for children (particularly for sensory issues), an ongoing point of contact for information and advice for parents, and child mental health support. This greater specificity may be reflective of increased recognition of sensory processing disorder and demand for mental health services in recent years.^46^, ^47^ Around a quarter of all CAMHS referrals in England are currently rejected, although there are substantial regional differences,^48^ and mental health support for children with autism is identified nationally as a key gap in service provision.^49^ In line with this, parents in this study reported difficulties gaining access to mental health services for their child due to high referral thresholds, with requests for support rejected even where children had exhibited self‐harm behaviours.

### 4.1 Strengths and limitations

The use of a visual timeline alongside qualitative interviews enabled rich data to be gathered, providing a detailed insight into parents' experiences of support at different stages of their child's journey with autism. The relatively large sample size (diverse in terms of child age, location and relative deprivation) allowed a range of experiences to be explored. However, although participants were from three regions of England and Wales and a mix of rural and urban localities, most were located in Wales and no inner‐city areas or regions of Northern Ireland or Scotland were included. In addition, few participants were from ethnic minority backgrounds‐ a factor that could significantly affect access to support,^50^ thus limiting the applicability of findings. Most interview participants were mothers of children with autism, some of whom described support from ‘other Mums’ as being vital. Therefore, it would be interesting to investigate the support experiences of fathers in more depth. Exploring children's own experiences, and experiences of siblings, would also provide an important (and currently little explored) perspective on the value and utility of different forms of support. As interview participants were parents/carers of children enroled in a randomized controlled trial of sensory integration therapy, those whose children received the therapy experienced additional, specialized support. To mitigate this, this interview study focused solely on the usual care families experienced outside the trial. Recruitment to the trial was via health, social services or education professionals, or self‐referral, therefore families in contact with support services and those seeking additional help for their child are likely to have been over‐represented in this interview study.

### 4.2 Recommendations for policy and practice

In terms of the period before autism diagnosis, training for professionals working with families (such as health visitors, psychologists and speech and language therapists) should raise awareness of the signs of autism, to improve early recognition and support, as recommended by the National Institute for Health and Care Excellence.^51^ Ensuring this support is provided in a nonjudgemental way may help reduce barriers to parents accessing statutory services. Following diagnosis, a clear pathway of support should be set out in local and national policy, to facilitate equitable access to services, regardless of families' resources and locality. Longer term, it is important that parents are signposted to ongoing sources of support in the community, such as local disability and parent groups, as these are highly valued by those who utilize them. Children should also have access to support from professionals specializing in autism. In particular, there is a need for timely mental health provision tailored to the needs of children with autism.

## 5 CONCLUSION

This study provides an in‐depth insight into experiences of support for children with autism and sensory processing difficulties. Provision is varied, meaning services are not equitably accessible in all areas or to all families. Vital support is provided via voluntary organizations and other parents, while the dismissal of children's needs by professionals is a key barrier to accessing statutory services. It would be useful for future research to explore the impact of ethnicity on access to support, as well as the perspectives of siblings, fathers and children with autism themselves. Findings emphasize the need for a clear pathway of support following diagnosis, autism‐specific training for professional service providers, signposting to ongoing local support groups and specialist support tailored to the needs of children with autism, particularly in relation to mental health.

## CONFLICTS OF INTEREST

The authors declare no conflicts of interest.

## AUTHOR CONTRIBUTIONS

*Conceptualization*: Sue Delport, Rachel McNamara, Monica Busse, David Gillespie, Elizabeth Randell, Lucy Brookes‐Howell, Alka S. Ahuja, Anne M. McKigney. *Data curation*: Rhys Williams‐Thomas. *Formal analysis*: Sarah Milosevic, Lucy Brookes‐Howell. *Funding acquisition*: Sue Delport, Rachel McNamara, Monica Busse, David Gillespie, Elizabeth Randell, Lucy Brookes‐Howell, Alka S. Ahuja, Anne M. McKigney. *Investigation*: Sarah Milosevic. *Methodology*: Lucy Brookes‐Howell, Sarah Milosevic. *Project administration*: Elizabeth Randell, Rhys Williams‐Thomas. *Supervision*: Lucy Brookes‐Howell, Rachel McNamara, Monica Busse. *Validation*: Lucy Brookes‐Howell. *Writing – original draft*: Sarah Milosevic. *Writing – review and editing*: Sarah Milosevic, Lucy Brookes‐Howell, Elizabeth Randell, Rhys Williams‐Thomas, Sue Delport, Monica Busse, David Gillespie, Alka S. Ahuja, Anne M. McKigney, Eleni Glarou, Rachel McNamara.

## Data Availability

The datasets generated and analysed during the study cannot be shared as participant confidentiality could be compromised if full interview transcripts were released.
